# Mastalgia: Prevalence at a Sub-Saharan African Tertiary Hospital

**DOI:** 10.1155/2014/972726

**Published:** 2014-09-30

**Authors:** T. Makumbi, M. Galukande, A. Gakwaya

**Affiliations:** ^1^Department of Surgery, College of Health Sciences, Makerere University, P.O. Box 7072, Kampala, Uganda; ^2^Department of Surgery, Mulago National Referral Hospital, Kampala, Uganda

## Abstract

*Introduction*. Mastalgia is a common breast condition among women referred to breast clinics worldwide. Whereas the prevalence is known in the Western world and Asia, the prevalence of the disease is unknown in many African countries. The aim of this study therefore was to determine the prevalence and describe factors associated with mastalgia among women attending a tertiary hospital in sub-Saharan Africa. *Methods*. A cross-sectional study was done in Kampala, Uganda. Mastalgia was defined as self-reported breast pain (unilateral or bilateral) for a period not less than two months. A pretested questionnaire was used to collect the data and statistical analysis was performed using SPSS version 11. Ethical approval was obtained. *Results*. Out of the 1048 women who presented to the breast clinic during the study period, 168 (16%) were diagnosed with mastalgia in the absence of breast cancer. Noncyclical and cyclical mastalgia were 22/168 (13%) and 5/168 (3%), respectively. The onset of noncyclical category as compared to the cyclical type of mastalgia was observed to manifest before 24 years of age (*P* = 0.006). *Conclusion*. Mastalgia was a common condition among women in this sub-Saharan African setting as is elsewhere. The early onset mastalgia in this sub-Saharan African study requires further exploration for determination of its risk factors.

## 1. Introduction

Mastalgia or mastodynia is breast pain and a recognized organic benign breast disease [1, 2]. Breast pain is the commonest breast symptom among women presenting to the outpatient department. It is reported by more than half of the female population during the reproductive years [3, 4]. It causes a certain degree of anxiety leading to repeated investigations and to some degree disturbs a woman's life style [5]. However, there is paucity of data about mastalgia in sub-Saharan Africa in general and Uganda in particular. Though mastalgia was originally considered a Western affliction, published data show that the disease is also prevalent in other parts of the world [5]. The aim of this study was to establish the prevalence and describe the clinical pattern and factors influencing mastalgia in women at a tertiary hospital breast clinic.

## 2. Methods 

### 2.1. Study Design

This cross-sectional study was carried out at the breast clinic of Mulago National Referral and University Teaching Hospital, Kampala, Uganda. A woman was defined as any female person who at least had experienced menstrual periods and had had breast pain for a period not less than 2 months. She should have undergone where appropriate a triple assessment screening test for the exclusion of breast cancer. Some amount of modularity (lumpiness) together with mastalgia is found in a normal population [6, 7]. Triple assessment being a multidisciplinary investigation of a breast mass by a clinician, radiologist, and pathologist (if indicated) was done independently and is known to exclude breast cancer [8, 9].

### 2.2. Exclusion and Inclusion Criteria

All women with suspicious lesions were excluded plus those with known manic psychiatric syndromes, in addition to those who had surgical or physical breast trauma within 6 months. Each patient was allocated a breast clinic unique identifier to ease reference to specific data (and record keeping). Thereafter each patient was seen through the hierarchy of the clinic and subjected to a triple assessment as and when indicated.

### 2.3. Ethical Considerations

The study was approved by the Mulago Hospital and Makerere University School of Medicine Research and Ethics Committees. Written informed consent was obtained and confidentiality ensured for each subject. A female nurse was present in the room while carrying out the interview and physical examination. A coded questionnaire was administered for each of the participants and filled in privacy.

## 3. Results

**Figure 1:**
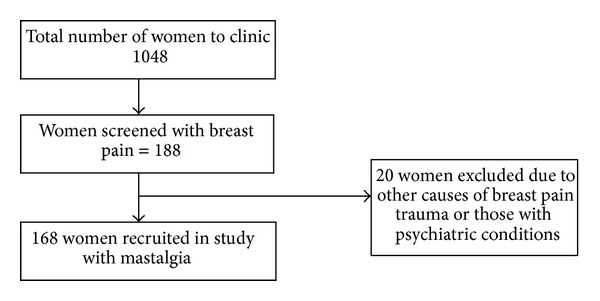
Women recruited with mastalgia from the breast clinic.

A total of 1048 women were seen at the breast clinic during the study period between December 2004 and May 2005. Out of these, 168 (16%) had mastalgia; see Figure 1. These 168 women distinctly exhibited a dual pattern distribution of which 82% had the noncyclical type while 18% had cyclical mastalgia. The youngest was 13 years and the oldest was 70 years. The mean age was 28.6 years. A majority of the women were in the 20–40-year-old group. Mastalgia showed no tribal or regional predilection though 39% of either category comprised young women (especially students). Most of the noncyclical cases were students (42%) while cyclical category mainly comprised (47%) semiskilled and self-employed women. The other characteristics are shown in Table 1. The mean and range for the duration of symptoms varied for either category of disease.

## 4. Discussion

We set out to determine the prevalence of mastalgia in women who attended our weekly breast clinic. We found that 16% of them had severe mastalgia, worrisome enough to cause them to come to the clinic. Most women with mastalgia will seek medical attention because of the fear of cancer or significantly affected quality of life. To our knowledge, no prior documentation of mastalgia in Uganda has been done.

Mastalgia, which was considered as a “Western affliction,” is no longer considered a rarity in other parts of the world [10]. More than 30% of women seen in breast clinics in Western countries have mastalgia [10]. The sample in this study accounted for a prevalence of 16% of all the women who were seen. The specific calculated prevalence of noncyclical and cyclical mastalgia was 13% and 3%, respectively, noncyclical one being most prevalent.

These two homogeneous and well-defined groups of noncyclical and cyclical mastalgia have been previously described [5, 11–13]. The actual number of women with mastalgia regardless of associated findings could therefore be much larger since only a few patients are able to present to the breast clinic at Mulago, a national referral hospital situated in the capital city, Kampala, for several reasons mostly socioeconomic.

The overall characteristics of either category of mastalgia were not pattern specific (Table 1) except for the comparatively early onset presentation of noncyclical mastalgia. There was also an observed though not statistically significant pain radiation to the arm in noncyclical type as compared to pain radiation to the axilla in cyclical category.

In the Postmenopausal women, noncyclic mastalgia was observed to be associated with early onset of breast pain and orientation along the breast/chest wall more so along the lateral wall as compared to cyclical mastalgia with a statistically significant late onset above 24 years (*P* = 0.006). Noncyclical mastalgia is usually unilateral and localized to a particular quadrant of the breast. Patients are usually older in their 40s and 50s and are often perimenopausal [14].

This study however showed no statistically significant correlation among BMI, parity, age at onset and age at presentation. In addition there was no correction between type of mastalgia and breast nodularity on the breast quadrant affected.

## 5. Study Limitations 

A breast pain dairy was not used and no hormonal profile for estrogen, progesterone, or prolactin levels was performed. Recall bias was a possibility in recounting duration of pain which could have led to either over- or underestimation. No investigations were done to exclude other recognized causes of nonbreast pain such as Tietze disease, peptic ulcer, or ischemic heart disease.

## 6. Conclusion

Mastalgia was a common symptom among women at this sub-Saharan African breast clinic. The early onset for the noncyclical type is unlike what has been previously known in literature and is worth further scrutiny.

## Figures and Tables

**Table 1:** Showing distribution of baseline characteristics.

Characteristics	Noncyclical (*n* = 138)	Cyclical (*n* = 30)
Mean age at presentation	28.1	30.7
Age at onset	25.7	27.6
BMI	23.3	24.1
Parity	1.8	1.5
Menopausal status		
Premenopausal	128	30
Postmenopausal	10	0
Duration of mastalgia		
Mean (weeks)	27.0	33.2
Laterality		
Unilateral	94	15
Left	48	10
Right	46	5
Bilateral	44	15
Site of pain		
Breast wall (chest wall)	52	10
Lateral	34	7
Medial	18	3
Breast quadrants	87	20
Less than 2 quadrants	49	8
Greater than 2 quadrants	38	12
Breast nodularity	59	15
Localized	49	10
Diffuse	10	5
